# Excision of a Large Cervical Glandular Tumor

**Published:** 1871-04

**Authors:** J. Wistar Vance


### EXCISION OF A LARGE CERVICAL GLANDULAR TUMOR.

BY J. WISTAR VANCE, L. R. C. S. & P. E.

Mary J—— came under my notice July 18th, 1870, suffering with a large swelling on the right side of the neck. The father of the child states that he noticed the tumor two years ago, and that it had been gradually increasing in size until lately, when it suddenly began to grow very fast, he thought that her memory was becoming affected. She seemed careless, stupid and indifferent of late—complained also of difficulty in swallowing. On making an examination, I found an oval-shaped, circumscribed, and movable tumor, every portion being movable except the most dependent, which could not be reached, as it extended below the insertion of the sterno mastoid muscle. There was no pain referred to the tumor, and the long time it had existed, together with the general good health of the patient, left no doubt in my mind of its being a simple glandular tumor. The physicians who had previously attended her had used the ordinary means of discussion without perceptible benefit. I accordingly advised an operation, which was performed on July 12th. Dr. Cavolus A. Simpson administering chloroform, an eliptical incision was made, extending from the martoid process of the temporal bone to a point opposite the sterno clavicular articulation, including a strip of skin about one-half inch in breadth. The skin, superficial fascia and platysma myoides having been cut through, the external jugular vein was carefully detached and held to one side by an assistant. The posterior fibres of the sterno martoid were next divided, and the tumor brought fully into view. It was found to have a great number of attachments to the surrounding parts, and was pressing against the great vessels of the neck, to the sheath of which it was also firmly attached. The lower edge of the tumor passed beneath the clavicle. The tumor was carefully removed, the handle of the knife being principally used to free it from its most dangerous attachments. Several bleeding vessels were secured by means of carbolized silk ligatures, the wound carefully washed out with cold water, and then closed with silver sutures; after which the wound was thoroughly syringed out with a solution of carbolic acid, (1 to 40) and carefully dressed according to Professor Lister's method. No oozing of any consequence took place afterwards. She complained immediately after the operation of great thirst. A small quantity of water was allowed at short intervals. Very soon violent vomiting and retching came on, which was relieved by the administration of one minim of dilute hydrocyanic acid. At bed time, 15 grains of chloral hydrat was given, which secured a comfortable night's rest. On the following morning she took some milk, with lime water and a little bread. Had no fever.

On the 3d day after the operation, the wound was found healed throughout, with the exception of the point where the ligatures were hanging out. A little pouting was noticed immediately below the ligatures. On making an examination to find the cause, a small silk thread was discovered lying between the edges of the wound, which, being removed, caused the pouting to disappear. The sutures were all removed on the 3d day. The child was up in eight days running about the house, though the last ligature did not come away till the 22d day.

The tumor was three inches in diameter, six inches in length, and weighed one pound.

